# Supplementary figures and images for: Identification of differentially expressed key genes between glioblastoma and low-grade glioma by bioinformatics analysis
Source: PeerJ. 2019 Mar 7;7:e6560. doi: 10.7717/peerj.6560 (PMC6409090; doi:10.7717/peerj.6560)

Figure S1

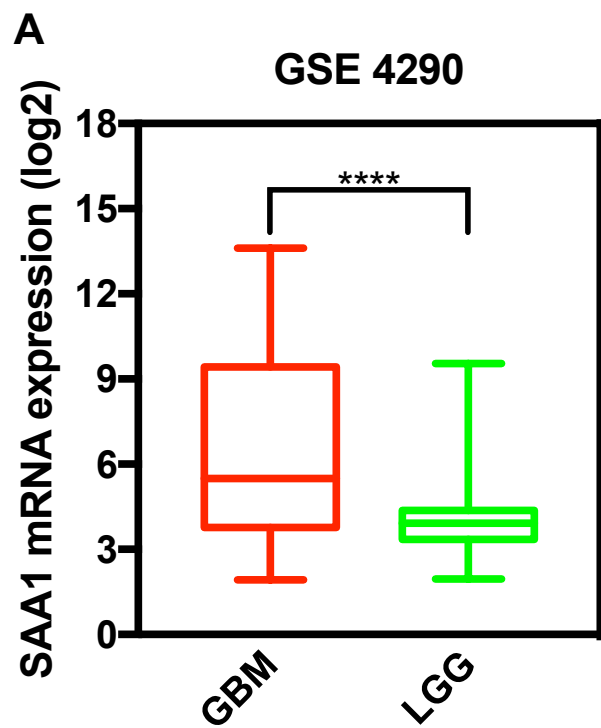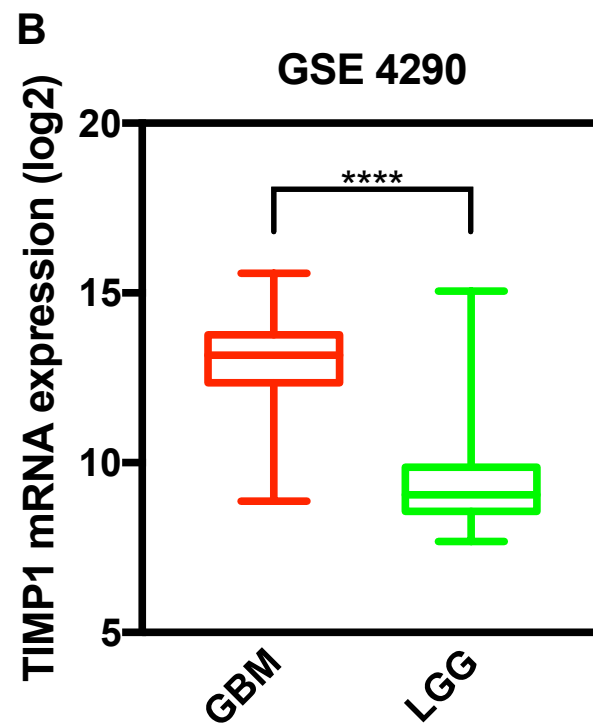

Supplement: Supplemental Information 1 [file peerj-07-6560-s001.pdf]

Figure S2

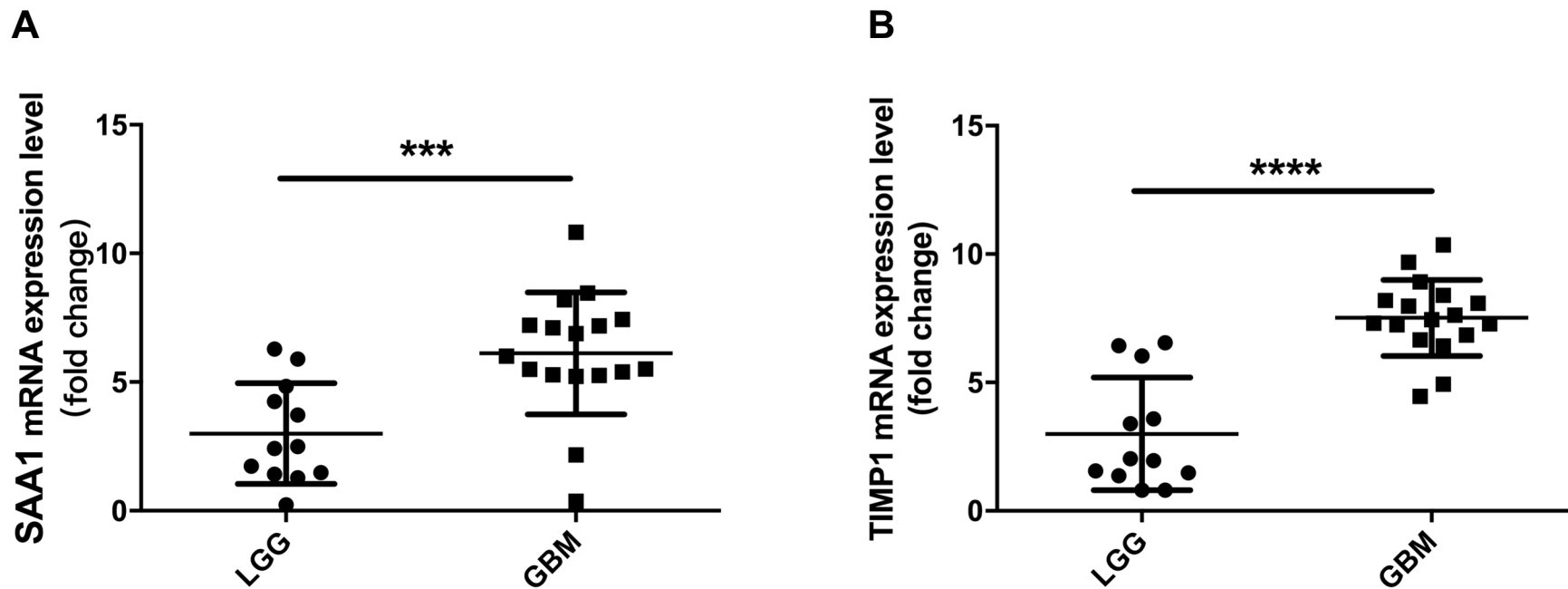

Supplement: Supplemental Information 2 — Figure S2: (A) SAA1 significantly increased in glioblastomas; (B) TIMP1 significantly increased in glioblastomas. [file peerj-07-6560-s002.pdf]
