# Supplementary material for: Identification of differentially expressed key genes between glioblastoma and low-grade glioma by bioinformatics analysis
Source: PeerJ. 2019 Mar 7;7:e6560. doi: 10.7717/peerj.6560 (PMC6409090; doi:10.7717/peerj.6560)
Supplement: Supplemental Information 3 — LGG: low grade glioma; GBM: glioblastoma; M: male; F: female; *Age at diagnosis was calculated from date of birth to date of surgery. [file peerj-07-6560-s003.docx]

**Table S1** Demographic data from patients analyzed in this study

| **Total of cases** | **Histology** | **Mean age at diagnosis (years)*** | **Gender** |
| --- | --- | --- | --- |
| 12 | LGG | 45.23 ± 10.82 | 5F, 7M |
| 17 | GBM | 53.00 ± 11.62 | 9F, 8M |

*LGG*: low grade glioma; *GBM*: glioblastoma; *M*: male; *F*: female; *Age at diagnosis was calculated from date of birth to date of surgery.
